# Non-steroidal or opioid analgesia use for children with musculoskeletal injuries (the No OUCH study): statistical analysis plan

**DOI:** 10.1186/s13063-020-04503-y

**Published:** 2020-09-03

**Authors:** Anna Heath, Maryna Yaskina, Gareth Hopkin, Terry P. Klassen, Christopher McCabe, Martin Offringa, Petros Pechlivanoglou, Juan David Rios, Naveen Poonai, Samina Ali, on behalf of the KidsCAN PERC Innovative Pediatric Clinical Trials No OUCH Study Group, Dennis Cote, Garth Meckler, Mark Roback, Anupam Kharbanda, Eyal Cohen, Lise Nigrovic, Geert W. ‘t Jong, Samina Ali, Naveen Poonai, Manasi Rajagopal, Lawrence Richer, Jennifer Thull-Freedman, Patrick McGrath, Timothy A. D. Graham, Lisa Hartling, Serge Gouin, Antonia Stang, Scott Sawyer, Maala Bhatt, Marie Christine Auclair, Kelly Kim, Lise Bourrier, Lauren Dawson, Kamary Coriolano DaSilva, Christopher McCabe, Gareth Hopkin, Jeff Round, Andy Willan, Maryna Yaskina, Anna Heath, David Rios, Martin Offringa, Petros Pechlivanoglou, Eleanor Pullenayegum, Pamela Marples, Rick Watts, Terry Klassen, Tannis Erickson, Brendon Foot, Serena Hickes, Kurt Schreiner, Julie Leung, Amy Drendel

**Affiliations:** 1 Child Health Evaluative Sciences, The Hospital for Sick Children, Toronto, Ontario Canada; 2 Dalla Lana School of Public Health, University of Toronto, Toronto, Ontario Canada; 3 Department of Statistics, University College London, London, UK; 4 Women and Children’s Health Research Institute, University of Alberta, Edmonton, Alberta Canada; 5 Institute of Health Economics, Edmonton, Alberta Canada; 6 University of Manitoba, Winnipeg, Manitoba Canada; 7 Children’s Hospital Research Institute of Manitoba, Winnipeg, Manitoba Canada; 8 Institute of Health Policy, Management and Evaluation, University of Toronto, Toronto, Ontario Canada; 9 Division of Neonatology, The Hospital for Sick Children, University of Toronto, Toronto, Ontario Canada; 10 Departments of Paediatrics, Internal Medicine, Epidemiology & Biostatistics, Schulich School of Medicine & Dentistry, Western University, London, Ontario Canada; 11 Children’s Health Research Institute, London, Ontario Canada; 12 Department of Pediatrics, Faculty of Medicine & Dentistry, University of Alberta, Edmonton, Canada; 13 Women and Children’s Health Research Institute, Edmonton, Alberta Canada

**Keywords:** Statistical analysis plan, Musculoskeletal injury, Pain management, Emergency department, Patient preference, Opioids, Analgesia

## Abstract

**Background:**

Pediatric musculoskeletal injuries cause moderate to severe pain, which should ideally be addressed upon arrival to the emergency department (ED). Despite extensive research in ED-based pediatric pain treatment, recent studies confirm that pain management in this setting remains suboptimal. The No OUCH study consist of two complementary, randomized, placebo-controlled trials that will run simultaneously for patients presenting to the ED with an acute limb injury and a self-reported pain score of at least 5/10, measured via a verbal numerical rating scale (vNRS). Caregiver/parent choice will determine whether patients are randomized to the two-arm or three-arm trial. In the two-arm trial, patients will be randomized to receive either ibuprofen alone or ibuprofen in combination with acetaminophen. In the three-arm trial, patients can also be randomized to a third arm where they would receive ibuprofen in combination with hydromorphone. This article details the statistical analysis plan for the No OUCH study and was submitted before the trial outcomes were available for analysis.

**Methods/design:**

The primary endpoint of the No OUCH study is self-reported pain at 60 min, recorded using a vNRS. The principal safety outcome is the presence of any adverse event related to study drug administration. Secondary effectiveness endpoints include pain measurements using the Faces Pain Scale-Revised and the visual analog scale, time to effective analgesia, requirement of a rescue analgesic, missed fractures, and observed pain reduction using different definitions of successful analgesia. Secondary safety outcomes include sedation measured using the Ramsay Sedation Score and serious adverse events. Finally, the No OUCH study investigates the reasons given by the caregiver for selecting the two-arm (Non-Opioid) or three-arm (Opioid) trial, caregiver satisfaction, physician preferences for analgesics, and caregiver comfort with at-home pain management.

**Discussion:**

The No OUCH study will inform the relative effectiveness of acetaminophen and hydromorphone, in combination with ibuprofen, and ibuprofen alone as analgesic agents for patients presenting to the ED with an acute musculoskeletal injury. The data from these trials will be analyzed in accordance with this statistical analysis plan. This will reduce the risk of producing data-driven results and bias in our reported outcomes.

**Trial registration:**

ClinicalTrials.gov NCT03767933. Registered on December 7, 2018.

## Background

Children who visit the emergency department (ED) after a musculoskeletal (MSK) injury often suffer moderate to severe pain [1, 2]. EDs, particularly the triage area, are usually the initial point of contact for children with MSK injuries and are therefore an ideal setting in which to manage pain by administering analgesia. Despite a substantial body of research in the area of pediatric pain, pain management within the ED is still suboptimal [3–5].

The most commonly used pharmacologic options for treating acute MSK pain in children are ibuprofen, acetaminophen, or opioids [3, 4, 6–8]. Despite this, the current evidence does not allow physicians or researchers to determine an analgesic strategy that offers an effective pain management strategy for patients with MSK injury. Primarily, this is because there are limited studies investigating the effectiveness of combination therapy, even though monotherapy offers inadequate pain management in around 50% of cases [9–12]. Thus, the Non-Steroidal or Opioid Analgesia Use for Children with Musculoskeletal Injuries study (the No OUCH Study) aims to collect evidence on the relative effectiveness of ibuprofen alone compared to two combination therapies; ibuprofen plus acetaminophen or hydromorphone.

There has recently been an effort to reduce opioid prescriptions in children [13, 14] due to concern about the potential harm from opioids and awareness of “the opioid crisis” in North America. Primarily, these concerns are based on the use of opioids in adults [15], rather than direct evidence on their safety and effectiveness profiles in children. Clinicians have become reticent to prescribe opioids to children and caregivers are increasingly less willing to accept them [5]. Therefore, the No OUCH study has been designed to understand and respect caregiver/parent preferences on opioid use.

The No OUCH study will utilize a novel preference-informed complementary trial design to assess the effectiveness and safety of ibuprofen, ibuprofen plus acetaminophen, and ibuprofen plus hydromorphone as a treatment for MSK injury-related pain in patients presenting to the ED. This design consists of two phase 2, multi-center, randomized, double blind, placebo-controlled trials that will be run simultaneously with the same study team, outcome definitions, and data management systems. The study protocol has been submitted separately [16]. This article outlines the statistical analysis plan (SAP) for the No OUCH study; it has been submitted for publication without knowledge of the study outcomes.

### Primary objective

The primary aim of the No OUCH study is to investigate two hypotheses. Firstly, we hypothesize that a combination of oral ibuprofen and oral acetaminophen has a greater analgesic effect than ibuprofen alone. Additionally, we hypothesize that a combination of oral ibuprofen and oral hydromorphone will have greater analgesic efficacy than ibuprofen alone or a combination of oral ibuprofen and oral acetaminophen. To investigate this, we will enroll a patient population that is aged between 6 and 17 years and presents at the ED with a limb injury that is less than 24 h old with a self-reported pain score at least 5 out of 10, using a verbal Numerical Rating Scale (vNRS) [17, 18].

## Methods/design

### Design and setting

The No OUCH study will use a preference-informed complimentary trial design that allows caregivers/parents to decide whether to enroll their child in a three-arm trial or two-arm trial. The three-arm trial (Opioid trial) includes the comparator ibuprofen plus hydromorphone alongside ibuprofen plus acetaminophen and ibuprofen alone. The two-arm trial (Non-Opioid) allows caregivers/parents to avoid the ibuprofen plus hydromorphone comparator. These two randomized, double-blind, placebo-controlled trials will be run simultaneously across six Canadian tertiary care pediatric EDs. The inclusion/exclusion criteria, study endpoints, and personnel will be identical across the two trials and we will consider whether a single treatment effect estimate across the two trials is appropriate.

### Study protocol development and conduct

The No OUCH study was registered on December 7, 2018, at ClinicalTrials.gov (trial registration number NCT03767933). The ethics committee at each participating site approved the study protocol prior to study implementation. The study is also regulated and monitored by Health Canada and is being conducted according to their recommended best practices. Consent will be obtained from all caregivers/parents and assent from participating children, where applicable. The No OUCH study resides within the KidsCAN-PERC iPCT network [19], a Canadian trials network with centralized infrastructure for data management and trial oversight. Within this network, an independent data and safety monitoring board has been recruited to monitor the study processes with a focus on the safety of the interventions.

### Randomization and data collection

Eligible patients will be approached for recruitment shortly after triage assessment, before analgesic medications might be offered by the triage nurse. Caregivers/parents will decide the trial in which they would like to participate. The Non-Opioid trial randomizes patients in a 1:1 ratio to either:

(i) Ibuprofen plus acetaminophen placebo OR
(ii) Ibuprofen in combination with acetaminophen.

The Opioid trial randomizes patients in a 1:1:1 ratio to either:

(i) Ibuprofen plus 2 placebos OR
(ii) Ibuprofen with acetaminophen plus oral hydromorphone placebo OR
(iii) Ibuprofen with oral hydromorphone plus acetaminophen placebo.

Within each trial, patients will be randomized, stratified by center, using block-randomization with variable block sizes. The relative recruitment rates for the two trials will be explored at an interim analysis (outlined below) and changes to the randomization/allocation ratio from 1:1 or 1:1:1 may be considered if there are recruitment issues for either of the trials.

The randomization order will be generated using a secure, online, centralized randomization tool housed at the Data Coordinating Centre (DCC) for the KidsCAN-PERC iPCT network, which is hosted at the Women and Children’s Health Research Institute (WCHRI) at the University of Alberta [20]. All research data collected from the study participants will be stored in the REDCap electronic data capture system [21]. The database is housed by the DCC in a secure data server within the WCHRI.

### Primary outcome

The primary outcome across both the No OUCH study is the self-reported pain score, measured by a 0–10 vNRS at 60 min after study drug administration. This vNRS has a minimally clinically important difference of 1.5 [22] and has been validated for pediatric pain [17, 23].

### Secondary outcomes

#### Efficacy outcomes

The No OUCH study will include seven secondary efficacy outcomes. Firstly, we will record which children achieve two alternative definitions of effective analgesia, a vNRS pain score of less than 3 out of 10, based on the WHO definition of mild pain [24], and a pain reduction of at least 2, known as “Patient Satisfaction with Analgesia” [22], at 60 min following the administration of the study drug. We will also investigate the difference in pain scores between the treatment arms in the two trials at 30, 60, and 120 min after the treatment administration, as well as at the time of the medical examination and at X-ray. We will also investigate whether children required rescue analgesia in the 60 min following the administration of the study medication. We will explore the length of stay for patients in the ED and the length of time to effective analgesia (a vNRS pain score of less than 3). Finally, we will record the children’s self-reported pain intensity using a visual analog scale and the Face Pain Scale-Revised (FPS-R) at all study times [25].

#### Safety outcomes

The principal safety outcome will be the incidence of any adverse events related to study drug administration. Additionally, we will record whether a participant experiences a serious adverse event during the study period and what types of adverse events they experienced. Finally, we will record the Ramsay Sedation Score (RSS) [26] for children in the study. The RSS will be dichotomised and any patient with a score between 1 and 3 will be deemed “over-sedated” as over-sedation is a potential risk with any opioid administration. To investigate whether appropriate pain management leads to issues in diagnosis, we will investigate the proportion of missed fractures or dislocations, as reported on follow-up survey.

#### Outcomes related to trial selection

The structure of the No OUCH study allows us to investigate the reasons for selecting the Opioid or Non-Opioid trial. We will determine the reasons that caregivers/parents selected the Opioid or Non-Opioid trial, and their self-reported satisfaction with the pain relief and comfort at home using a 5-point Likert scale (c.f. supplementary material). Finally, at ED discharge, we will record the analgesic that the physician would have preferred to give the patient, if the patient were not enrolled in the study.

### Sample size calculation

The No OUCH study comprises two concurrent trials with a sample size calculation undertaken separately for each trial. The sample size for the Opioid Trial is 105 patients per arm or 315 in total. For the Non-Opioid Trial, the sample size is 85 patients per arm or 170 in total. Thus, the total recruitment for the No OUCH study will be a minimum of 485 patients. To account for missing data for the primary outcome due to early withdrawal, the study will over-recruit by approximately 10%, for a target recruitment of 540 patients. This sample size was calculated assuming a two-sided level of 0.05, a power of 0.95, a minimally clinically important difference of 1.5 on the vNRS and an estimate of the standard deviation of the difference of 2.7 [22]. We used a Bonferroni correction to adjust for the three treatment comparisons in the Opioid trial.

Based on prior survey data, we are not expecting the two No OUCH trials to complete recruitment at the same time. Thus, we aim to keep recruitment open for both trials until both trials reach the required number of participants. This could require over-recruitment in one of the two trials and therefore the total recruitment for the No OUCH study could exceed 540. This over-recruitment is required to ensure that the caregiver/parent preference aspect of the No OUCH study is respected throughout. To avoid significant over-recruitment, we will monitor the recruitment rate of the two trials. If we face significant over-recruitment for the Non-Opioid trial, then the study team, in consultation with the Data Safety Monitoring Board (DSMB), will consider randomizing a larger proportion of patients to the hydromorphone plus ibuprofen arm within the Opioid trial to ensure 105 patients are recruited for this comparator. If the relative recruitment rate to the two trials is very highly skewed, we will consider stopping the No OUCH study when the first, rather than the second, trial completes recruitment and adjusting our hypotheses accordingly.

### Interim analyses and stopping guidance

A DSMB with six participants has been created for the No OUCH study to protect patients and advise the study team. Biannually, the DSMB will be provided with a summary and list of the protocol deviations and adverse events across all trial participants. The DSMB can also request a summary broken down by treatment group and trial, alongside the associated *p* values. The DSMB will have sole control over whether to stop one or both the No OUCH trials for safety reasons. The No OUCH study will not consider early stopping for efficacy or futility, and thus, no interim analysis is planned for the outcome measures. No statistical adjustments are made for interim analyses of the efficacy outcome data.

There will be an initial interim analysis of the relative recruitment rate into the Opioid and Non-Opioid trials to ensure timely completion of both trials. This interim analysis of the recruitment rate will take place after 100 patients have been enrolled across the No OUCH study. Depending on the relative recruitment rates across the two trials at this interim analysis, we will either continue to monitor the recruitment rate, change the randomization ratio in one of the trials, or stop the No OUCH study once one trial is completed. Any change in the sample size or randomization ratio will be carefully evaluated to reduce the risk of bias and maintain sufficient power.

## Statistical analysis plan

### Statistical principles

Analyses will be performed according to the intention-to-treat principle. For all relevant parameters, 95% confidence intervals will be presented. Broadly, if an outcome is subjected to formal statistical testing, we will declare significance at the 5% level and use a Bonferroni-Holm correction to adjust the analysis for multiple comparisons when required. When descriptive statistics are used, we will present means, standard deviations, medians, and interquartile ranges for continuous variables and summarize discrete variables with frequency distributions. All analyses will be performed in either SAS [27] or R [28]. As the endpoints for the two trials are the same, our primary analysis will consider whether a joint analysis across the two trials is valid, both from a clinical and statistical perspective. Thus, the statistician involved in analyzing the trial data will not be blinded to the combination of ibuprofen with hydromorphone within the Opioid trial. However, the statistician will remain blinded to the other two arms throughout the analysis.

### Timing of outcomes and analysis

The majority of the outcomes for the No OUCH study will be collected while the patient is in the ED for their enrolment visit. Outcomes related to patient response and caregiver/parent satisfaction with the intervention will be collected over the phone or by email. These outcomes will be collected within 1–3 days of discharge from the ED and again at 1–2 weeks post-discharge. The final analysis for the No OUCH study will be undertaken once every patient has reached 2 weeks after enrollment and the database has been cleaned and locked. The analysis will be performed by a statistician who did not assess the trial outcomes.

### Handling of missing data

As the primary endpoint is assessed in the ED within 2 h of treatment allocation, we anticipate minimal missing data for the primary analysis. The study team will endeavor to collect the relevant data for all outcomes and therefore avoid missing data in the secondary or exploratory outcomes. Multiple imputation will be used to impute any missing data, including outcomes as required, in the cleaned and locked dataset [29].

### Patient flow

We will use a CONSORT 2010 flow diagram to provide details on the patient flow [30]. We will report the number of patients screened, the number of patients who met our trial inclusion criteria, and the number of patients ineligible for the No OUCH study based on the criteria outlined in the trial protocol [16], for both trials. The CONSORT diagram will also present how many patients were lost to follow-up and when they withdrew from the study. We will include the number and reasons for withdrawal and/or exclusion from analysis at each stage. Finally, we will provide descriptive statistics for the sex, age, eligibility criteria, and consent status of all screened patients across both trials.

### Protocol deviations

Within the No OUCH study, protocol deviations are defined as patients who:

(i) Are randomized but do not receive the allocated study intervention OR
(ii) Did not meet the inclusion criteria or met one of the exclusion criteria and are enrolled in the study OR
(iii) Receive the incorrect dose of the study medication.

A patient is said to have successfully adhered to the treatment if they complete the one-time administration of the allocated study drugs. As such, we are expecting high levels of adherence.

We will classify all protocol deviations before unblinding the treatment options. The raw number and the percentage of patients with protocol deviations and non-adherence will be summarized by treatment group. We will also include details of which type of protocol deviation was experienced. Percentages will be calculated using the number of patients in the intention to treat dataset. We will not use formal statistical testing to investigate protocol deviations.

### Baseline characteristics

For both trials, we will present the age, sex, injury type, injury location, and vNRS score at time of recruitment for both trials and all treatment options, including non-pharmacological strategies such as a splint. These characteristics will be presented using appropriate descriptive statistics.

### Analysis for the primary endpoint

The primary effectiveness analysis will compare the vNRS score at 60 min after study drug administration using linear mixed models to estimate the treatment effect while adjusting for the vNRS score at baseline and a site-specific random effect for each of the six participating sites. As the two No OUCH trials have the same primary endpoint and inclusion/exclusion criteria, we will consider whether it is appropriate to estimate a single pooled treatment effect across the two trials. We will only estimate a single treatment effect, if there is neither clinical nor statistical evidence that this should not be undertaken. To determine clinical rationale for a single pooled treatment effect, we will evaluate whether the baseline clinical characteristics across the two trials (including pain scores at baseline) indicate a difference between the two participant populations. This will be assessed before undertaking analysis for the primary outcome and will be based on clinical judgment defined using consensus among experts. The supplementary material outlines the method we will use to extract the clinical judgment.

If we determine that there is no clinical evidence of a difference across the two patient populations, we will then use a likelihood ratio test to determine whether there is statistical evidence of a difference in the treatment effect across the two trials. To perform this test, we will fit two nested linear mixed models for self-reported vNRS pain scores at 60 min, adjusting for vNRS pain score at baseline and a site-specific effect. The full model will include a treatment by trial interaction term while the reduced model will assume a constant treatment effect across the two trials.

If the likelihood ratio test suggests a superior fit for the full model at the 5% level, then data from the two trials will be analyzed separately. In this setting, two linear mixed models will be used to estimate a trial-specific treatment effect. These two models will both adjust separately for baseline vNRS score and site. If the full model does not provide a statistically superior fit at the 5% level, then the reduced model will be used to analyze the data and obtain single treatment effects for ibuprofen in combination with acetaminophen or hydromorphone. The family-wise error rate will be controlled when declaring significance for the primary endpoints, irrespective of whether a pooled a treatment effect is estimated or not.

### Analysis for secondary endpoints

If the analysis for the primary outcome provides a single pooled treatment effect, the analysis for the secondary outcomes will be pooled. Otherwise, we will analyze the trials separately for the secondary outcomes.

#### Efficacy outcomes

We will compare the proportion of children with a self-reported vNRS pain score of less than 3 out of 10 at 60 min (success) across treatment groups using a logistic mixed model, adjusted for site and baseline pain. We will also use a logistic mixed model to compare the proportion of children who require a rescue analgesic by 60 min, considered as a failure for the analgesic agent, across the different treatments. For both these outcomes, we will report the odds ratio and adjust for multiple comparisons. All other secondary outcomes for effectiveness will be summarized within each treatment group using the appropriate descriptive statistics.

#### Safety outcomes

We will compare the proportion of children with adverse events related to study drug administration using a logistic mixed model, adjusted for site. This analysis will not adjust for baseline pain. All other safety outcomes will be summarized using appropriate descriptive statistics.

#### Trial selection outcomes

All outcomes relating the caregiver/patient trial preference will be summarized using appropriate descriptive statistics. They will also be further explored in a qualitative substudy to illuminate the reasons for avoiding opioids.

### Subgroup analyses

We will perform two pre-planned exploratory subgroup analyses comparing the self-reported vNRS pain score at 60 min by age group, under and over or equal to 12 years, and by type of injury, fracture or soft tissue injury. These analyses will be undertaken using linear mixed models with an interaction term between treatment and subgroup. These analyses will be performed within each trial separately or pooled across the two trials depending on the analysis for the primary endpoint.

### Sensitivity analyses

If the two trial populations are analyzed together as the primary analysis, we will analyze the two trials separately as a sensitivity analysis. We will undertake the primary analysis considering self-reported vNRS pain score as an ordinal categorical variable, rather than a continuous variable. Finally, we will undertake an exploratory analysis from a Bayesian perspective for the primary outcome and all secondary outcomes that are subject to frequentist testing. This analysis will use minimally informative and informative priors. The posterior distributions for the parameters will be summarized using posterior means and 95% posterior credible intervals.

### Trial status

The No OUCH study was registered on December 7, 2018, at ClinicalTrials.gov and started recruitment at the Stollery Children’s Hospital on May 13, 2019. Recruitment is currently underway and is expected to complete around summer 2021. Before the data are analyzed, the database will be cleaned and checked for completeness, blinded to treatment allocation. The database will be locked and the analysis will be undertaken using the methods specified in this SAP.

## Supplementary information

**Additional file 1.** Pooling Guidelines

**Additional file 2.** 5-Point Likert Scale

## Data Availability

No datasets were used to develop this article as analysis was not undertaken. Thus, this consideration is not applicable.

## Abbreviations

MSK: Musculoskeletal
ED: Emergency department
DSMB: Data Safety Monitoring Board
RSS: Ramsay Sedation Score
vNRS: Verbal Numerical Rating Scale
FPS-R: Faces Pain Scale-Revised
SAP: Statistical analysis plan
No OUCH: Study of Non-Steroidal or Opioid Analgesia Use for Children with Musculoskeletal Injuries
WCHRI: Women and Children’s Health Research Institute
DCC: Data Coordinating Centre
