# Supplementary material for: Non-steroidal or opioid analgesia use for children with musculoskeletal injuries (the No OUCH study): statistical analysis plan
Source: Trials. 2020 Sep 3;21:759. doi: 10.1186/s13063-020-04503-y (PMC7469310; doi:10.1186/s13063-020-04503-y)
Supplement: Supplementary file 1 — Additional file 1. Pooling Guidelines [file 13063_2020_4503_MOESM1_ESM.docx]

**Supplementary Material**

**Pooling Guidelines for the No OUCH study**

There guidelines specify how clinical guidance will be sought to determine whether a single treatment effect for each of the comparisons in the trial, ibuprofen against acetaminophen/ibuprofen, ibuprofen against hydromorphone/ibuprofen and acetaminophen/ibuprofen against hydromorphone/ibuprofen, will be estimated from the No OUCH study. The trials will be analyzed separately if their baseline characteristics vary considerably, defined using clinical judgement, across the trials. The No OUCH study collect information on five baseline characteristics for the patients in the trial:

1. age
2. sex
3. injury type
4. injury location
5. baseline vNRS score

Clinical judgement will be used to assess whether these baseline characteristics are different across the two trials before undertaking the analysis for the outcomes. The following outlines the proposed methodology for eliciting this clinical judgement.

**Interim Analysis of Baseline characteristics**

The DSMB will meet biannually to receive reports on recruitment, safety events and protocol violations. The total target recruitment for the No OUCH study is 540. Thus, once recruitment has exceeded 268 across the two trials, we will extract the baseline characteristics for the participants in the two trials. These baseline characteristics will be presented to a panel of experts in the standardised format used for clinical manuscripts. Alongside this, two “pseudo-datasets” will be created where the patients in the No OUCH study are randomly assigned to the two trials and the baseline characteristics are summarised. The experts will be asked whether they would consider pooling these two populations for the three datasets. If these experts agree that all these populations can be subject to a pooled analysis, we will evaluate the baseline characteristics at trial completion. If these characteristics are similar to the interim analysis, we will proceed with only the formal testing procedure to determine whether the No OUCH analysis can be pooled.

If the experts are unconvinced that the analysis can be pooled, we will elicit guidelines to support our decision to estimate a single treatment effect by reaching out to Pediatric Emergency Research Canada (PERC) members. This will allow us to understand how acceptable the pooled analysis from the No OUCH study would be to clinicians.

**Final Analysis**

The final decision on whether to estimate a single treatment effect due to similarity between the baseline characteristics will be taken by the trial analysis team. The guidelines developed at the interim analysis will be used to support this decision. The baseline characteristics will be extracted from the database *prior* to data extraction on the primary outcomes. The decision to proceed with a likelihood ratio test to determine any statistical difference between the populations will be stated before extracting and analysing the outcome data.
