# Supplementary material for: Non-steroidal or opioid analgesia use for children with musculoskeletal injuries (the No OUCH study): statistical analysis plan
Source: Trials. 2020 Sep 3;21:759. doi: 10.1186/s13063-020-04503-y (PMC7469310; doi:10.1186/s13063-020-04503-y)
Supplement: Supplementary file 2 — Additional file 2. 5-Point Likert Scale [file 13063_2020_4503_MOESM2_ESM.docx]

**5-Point Likert Scale**

| How do you feel about the pain treatment provided by the medicines your child was given in the emergency department, as part of this study? | Very Satisfied |
| --- | --- |
|  | Somewhat Satisfied |
|  | Neutral |
|  | Somewhat Dissatisfied |
|  | Very Dissatisfied |
